# Association of high sensitive C-reactive protein with coronary heart disease: a Mendelian randomization study

**DOI:** 10.1186/s12881-019-0910-z

**Published:** 2019-11-06

**Authors:** Qian Zhuang, Chong Shen, Yanchun Chen, Xianghai Zhao, Pengfei Wei, Junxiang Sun, Yanni Ji, Xiaotian Chen, Song Yang

**Affiliations:** 1 Department of Cardiology, Affiliated Yixing People’s Hospital of Jiangsu University, Yixing, China; 2 Department of Epidemiology, School of Public Health, Nanjing Medical University, Nanjing, China; 3 Department of Clinical Epidemiology, Children’s Hospital of Fudan University, Shanghai, 201102 China

**Keywords:** Hs-CRP, *CRP* gene, CHD, Mendelian randomization analysis

## Abstract

**Objectives:**

Whether high sensitivity C-reactive protein (hs-CRP) has a causal effect on coronary heart disease (CHD) is unclear. This study investigated the causal effect of hs-CRP on CHD risk using Mendelian Randomization (MR) analysis.

**Methods:**

A total of 3802 subjects were recruited in the follow-up study. Linear regression model was used to evaluate the relationship between *CRP* polymorphisms and hs-CRP. Survival receiver operator characteristic curve method was used to explore the cut-off of hs-CRP on CHD incidence. Cox regression model was applied to detect the association of hs-CRP with CHD by calculating the hazard ratio (*HR*) and 95% confidence interval (*CI*). Rs1205 and rs876537 in *CRP* were selected as instrumental variables in MR analysis.

**Results:**

During a median follow-up time of 5.01 years, 98 CHD incidence was identified (47.03/10^4^ person-years). Hs-CRP was significantly increased among rs1205 and rs876537 genotypes with *r* values of 0.064 and 0.066, respectively. Hs-CRP 1.08 mg/L was identified as the cut-off value with a maximum value of sensitivity and specificity on prediction of CHD. Participants with ≥1.08 mg/L of hs-CRP has a higher risk of CHD incidence than that of participants with < 1.08 mg/L, the adjusted *HR* (95% *CI*) was 1.69 (1.11–2.60) with a *P* value of 0.016. No significant casual association was observed between hs-CRP and CHD with a *P* value of 0.777.

**Conclusions:**

The association between hs-CRP and CHD is unlikely to be causal, hs-CRP might be a predictor for incidence of CHD in general population.

## Background

Coronary heart disease (CHD) is one of the leading global cause of death and disability. The current situation of rapid aging in China makes CHD become the 4th leading cause of mortality [1]. The increased adiposity, type 2 diabetes mellitus (T2DM), hypertension, sedentary lifestyle, as well as genetic factors, are the well-known determinants of CHD [2].

Inflammation promotes endothelial cell damage and atherogenesis, is an important risk factor for CHD in clinical [3]. As a famous parameter of inflammation, C-reactive protein (CRP) is also considered a valuable predictor of CHD risk [4]. To meet the super-precision of clinical practice, the measurement of high sensitivity C-reactive protein (hs-CRP) is proposed for its better capacity in identifying the individuals at high risk for CHD [5]. Hs-CRP has commonly been used for cardiovascular diseases (CVDs) risk stratification. The elevated level of serum hs-CRP was significantly increased the risk of myocardial infarction [6]. Randomized clinical trials have observed that the use of statins in individuals with elevated hs-CRP was associated with a reduction in hs-CRP and a decreased risk of vascular events [7]. Even numerous of studies support that the hs-CRP is an established marker for future risk of CVDs, however, inconsistent findings have been reported on CHD [8, 9]. Therefore, it has considerable interest in establishing whether hs-CRP has a causal role in CHD.

Mendelian randomization (MR) studies utilize genetic variants (such as he-CRP) as instrumental variables (IVs) to investigate possible causal relationship between exposure and outcomes through an intermediate trait [10]. The dominant advantage of MR studies is that they are not as vulnerable to confounding and reverse causality [11, 12]. If the intermediate trait is causally linked to disease, then genetic variants influencing the trait should also influence disease risk. Indeed, MR has been regarded as nature’s analogue of randomized controlled trials, thus, it has also been used in cardiovascular research to explore novel potential etiologic mechanisms and enhance our understanding of current therapies [13, 14].

The aim of the current study was to identify common single nucleotide polymorphisms (SNPs) in *CRP* that influence hs-CRP levels, and use the concept of MR to improve understanding of the possible causal relationship of hs-CRP levels with CHD.

## Methods

### Study population

A total of 4222 participants were drawn from the community hypertension survey in Yixing city, China, 2009 [15]. After we excluded the individuals with a history of CHD (*n* = 50), elder participants (*n* = 94) and missing measurement of hs-CRP (*n* = 373), 3802 subjects were finally enrolled in the follow-up study.

To access the incidence of disease status in the follow-up study, both concentrated and household surveys were conducted. Data collected from the local hospitals, Centers for Disease Control and Prevention, community health service centers and social security center was further inspected to reduce the information bias. During a median follow-up time of 5.01 years, 98 new-onset CHDs were recorded.

All participants were interviewed and underwent physical examinations and laboratory tests. The demographic characteristics of the participants were obtained by trained research staff though a standard questionnaire. The weight, height and 3 blood pressure measurements were obtained from each participant by trained and certified observers according to a standard protocol.

Written informed consent was obtained from all participants. The research protocol was approved by the ethics committee of Nanjing Medical University and written informed consent was obtained from all subjects during epidemiological interviews.

### SNP selection

The *CRP* gene was located on chromosome 1q23.2 (Gene ID: 1401; NC_000001.11) and spans 2.3 kbps and contains 4 exons. We searched the SNPs covered *CRP* gene from the upstream 5 kb to the downstream 2 kb and selected tagging SNPs (tagSNPs) from the database of the Chinese Han population in Beijing, China of the International Hap MAP Project (HapMap Data Rel 24/phase II Nov08, on NCBI B36 assembly, dbSNPb126). All tagSNPs were selected with a minor allele frequency (MAF) ≥ 0.05 and linkage disequilibrium (LD) *r*^*2*^ ≥ 0.8. We also applied a functional candidate strategy to select potential functional SNPs on the bioinformatics effect prediction website (SNPINFO, https://snpinfo.niehs.nih.gov/). Finally, rs1205(C > T), rs1073715(C > T), rs876537(C > T) and rs2808630(C > T) were selected. The specific biological information was summarized in the Additional file 1: Table S1.

### Blood sampling

Blood samples were taken from participants after an over-night fasting (> 10 h), adding ethylenediamine tetraacetic acid (EDTA)-containing receptacles. The blood samples were centrifuged at 3500 rpm to separate the serum and stored at − 20 °C. Serum hs-CRP, total cholesterol (TC), triglycerides (TG), high-density lipoprotein cholesterol (HDL-C), low-density lipoprotein cholesterol (LDL-C) and glucose (GLU) were measured by an automatic biochemical analysis system of Siemens (Advia 1200, German).

Following recommendations from the American Centers for Disease Control and the American Heart Association [16], the participants with hs-CRP ≥1.0 mg/L and hs-CRP < 1.0 mg/L correspond to high risk group (HRG) and normal group (NG), respectively.

### SNP genotyping

DNA was extracted using a standard phenole-chloroform method. Genotyping was performed using the TaqMan allelic discrimination assay in 384-well plates on the platform of 7900HT Real-time polymerase chain reaction (PCR) System (Applied Biosystems, Foster City, CA). The primers and probes were designed using Primer Express Oligo Design software v2.0 (ABI PRISM). Genotyping results were determined using SDS 2.3 Allelic Discrimination Software (Applied Biosystems). Meanwhile, each plate was included blank samples as negative controls for the genotyping quality confirmation. The successful call rates of SNPs rs10737175, rs1205, rs2808630 and rs2966449 were 98.44, 98.31, 98.39 and 98.41%, respectively.

### Statistical analysis

Unpaired Student’s t-test was used to test the differences in of all the quantitative variables among groups presented as means ± standard deviation (SD). Chi-square (*χ*^*2*^) test was performed to compare the proportion of hypertension, T2DM, smoking and drinking between HRG and NG group. Linear regression model was used to analyze the correlation between the variations of *CRP* gene and hs-CRP. Besides, we used survival receiver operator characteristic curve (SROC) to explore the cut-off values of hs-CRP with CHD incidence. Cox proportional hazard regression model was applied to evaluate the association of *CRP* gene with CHD by calculating hazard ratio (*HR*) and 95% confidence interval (*CI*).

**Fig. 1:**
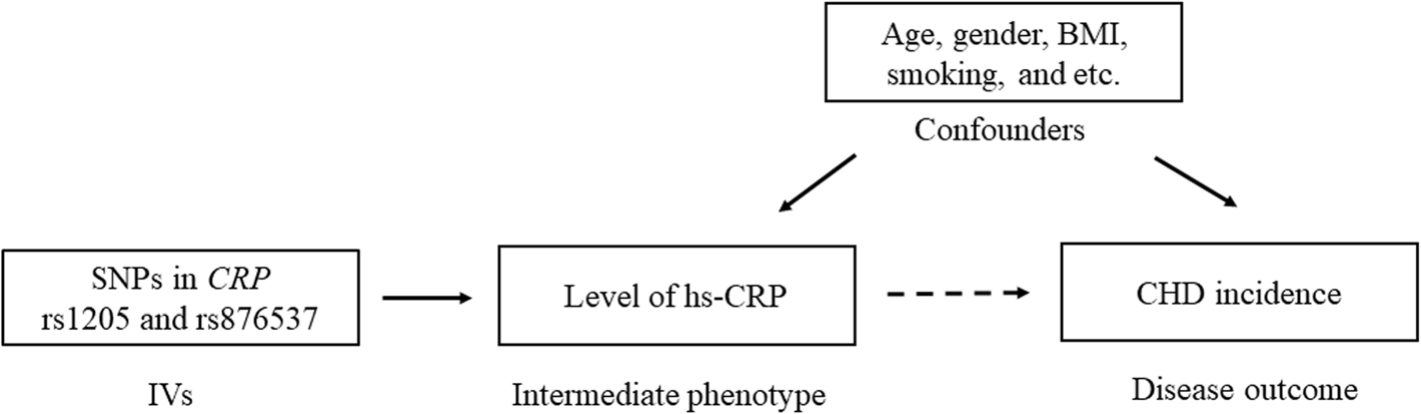
The basic principle of Mendelian Randomization analysis for the current study. BMI, body mass index; CRP, C-reactive protein; CHD, coronary heart disease; IVs, instrumental variables; SNP, single nucleotide polymorphisms

The MR analysis for exploring the causal effect of hs-CRP on the risk of CHD applying inverse-variance weighted (IVW) methods, where the SNPs are deemed as IVs. The basic principle of MR analysis for the current study was shown in Fig. 1. Effects are given as odds ratios (*OR*) and 95%*CI*. A two-tailed *P* value of 0.05 was defined as the cut-off for statistical significance. The SROC and MR analysis were undertaken using the R Packages of “survivalROC” and “Mendelian Randomization”.

## Results

### Demographic characteristics at baseline

The demographic characteristics of participants were summarized in Table 1. The age, BMI, SBP, DBP, TC, TG, LDL-C, GLU and proportion of hypertension in HRS were significantly higher than NG, whereas HDL-C, the proportion of T2DM, smoking and drinking were significantly lower (*P* < 0.05). Overall, these characteristics were adjusted as confounding factors in the multivariate regression analysis.

**Table 1:** Baseline characteristics for different hs-CRP groups

Variables	Group	HRG	NG	*t/χ* ^*2*^	
		*n* = 1695	*n* = 2107		*P*
Age		62.30 ± 10.84	58.62 ± 10.34	10.639	< 0.001
Gender, n (%)	Male	652(38.47)	920(43.66)	10.546	< 0.001
	Female	1043(61.53)	1187(56.34)		
Drinking, n (%)	Yes	346(20.41)	499(23.68)	5.811	0.016
	No	1349(79.59)	1608(76.32)		
Smoking, n (%)	Yes	383(22.59)	556(26.39)	7.264	0.007
	No	1312(77.41)	1551(73.61)		
BMI (kg/m^2^)		24.99 ± 3.56	23.56 ± 3.11	13.267	< 0.001
SBP (mmHg)		135.12 ± 15.78	132.18 ± 15.37	5.791	< 0.001
DBP (mmHg)		83.58 ± 8.47	83.09 ± 8.58	1.753	0.08
Hypertension, n (%)	Yes	914(53.92)	918(43.57)	40.336	< 0.001
	No	781(46.08)	1189(56.43)		
TC(mmol/L)		4.96 ± 1.06	4.74 ± 1.01	6.407	< 0.001
TG (mmol/L)		1.87 ± 1.50	1.53 ± 1.31	7.242	< 0.001
HDL-C (mmol/L)		1.35 ± 0.32	1.39 ± 0.33	3.705	< 0.001
LDL-C (mmol/L)		2.77 ± 0.83	2.67 ± 0.77	3.909	< 0.001
GLU (mmol/L)		5.80 ± 2.02	5.49 ± 1.62	5.273	< 0.001
T2DM, n (%)	T2DM	222(13.10)	184(8.73)	35.962	< 0.001
	IFG	418(24.66)	426(20.22)		
	No	1055(62.24)	1497(71.05)		

*BMI* body mass index, *DBP* diastolic blood pressure, *GLU* glucose, *HDL-C* high-density lipoprotein cholesterol, *HGR* high risk group, *IFG* impaired fasting glucose, *LDL-C* low-density lipoprotein cholesterol, *NG* normal group, *SBP* systolic blood pressure, *TC* total cholesterol, *TG* triglyceride, *T2DM* Type 2 diabetes mellitus

### Association of *CRP* polymorphisms with CHD incidence

During a median follow-up time of 5.01 years, 98 CHD incidence was identified with an incidence density of 47.03/10^4^ person-years. No significant associations were observed between each of the four SNPs with CHD in the whole population. After adjusted for age, gender, BMI, HDL-C, LDL-C, TC, TG, smoking, drinking, hypertension and T2DM, the results were still not significant (Table 2). Stratification analysis by age (55 years), gender, smoking and drinking status were further conducted, however, no significant associations were found between *CRP* and CHD (Additional file 1: Table S2).

**Table 2:** Association analysis of *CRP* polymorphisms with CHD incidence

SNP	Genotype	N	Person-years	Incidence density (/10^4^ Person-years)	*HR* (95% *CI*)^a^		
					Additive model	Dominant model	Recessive model
rs10737175	CC	56	12,085.13	46.34	1.03(0.75–1.41)	1.00(0.68–1.48)	1.19(0.55–2.57)
	CT	35	7514.93	46.57	*P* = 0.873	*P* = 0.995	*P* = 0.668
	TT	7	1246.34	56.16			
rs1205	CC	34	6872.61	49.47	1.03(0.78–1.36)	0.90(0.60–1.35)	1.26(0.79–2.03)
	CT	43	10,196.32	42.17	*P* = 0.851	*P* = 0.615	*P* = 0.338
	TT	21	3756.4	55.9			
rs2808630	TT	71	14,371.91	49.4	0.81(0.55–1.19)	0.80(0.52–1.23)	0.64(0.15–2.72)
	TC	24	5901.37	40.67	*P* = 0.274	*P* = 0.300	*P* = 0.546
	CC	3	563.1	53.28			
rs876537	CC	34	6849.88	49.64	1.03(0.78–1.36)	0.91(0.61–1.37)	1.24(0.77–1.99)
	CT	43	10,189.35	42.2	*P* = 0.848	*P* = 0.659	*P* = 0.374
	TT	21	3802.17	55.23			

Additive model: wild type vs heterozygote vs mutant

Dominant model: wild type vs heterozygote + mutant

Recessive model: wild type *+* heterozygote vs mutant

*HR* hazard ratio, *CI* interval confidence

^a^Adjusted for age, gender, BMI, HDL-C, LDL-C, TC, TG, smoking, drinking, hypertension and T2DM

### Association of hs-CRP with CHD

The incidence density of CHD for HRG and NG group were 71.08/10^4^ and 33.79/10^4^ person-years, respectively. The elevated hs-CRP was significantly increased CHD risk, the *HR* (95% *CI*) was 2.05 (1.36–3.09) with a *P* value of 0.001. But, the result was still not significant after adjusted for cofounders, the adjusted *HR* (95% *CI*) was 1.54 (0.99–2.36) with a *P* value of 0.051.

The SROC analysis showed that the highest sensitivity and specificity of cut-off point for hs-CRP to predict CHD incidence was 1.08 mg/L, the sensitivity and specificity were 60.2 and 59.1%, respectively (Additional file 1: Figure S1). Participants with ≥1.08 mg/L of hs-CRP has a higher risk of CHD incidence than that of participants with < 1.08 mg/L (74.92/10^4^ vs 32.97/10^4^ person-years), the adjusted *HR* (95% *CI*) was 1.69 (1.11–2.60) with a *P* value of 0.016. Detailed results were displayed in Table 3.

**Table 3:** Association analysis of hs-CRP with CHD in the follow-up study

Variables	Group	N	person-years	Incidence density (/10^4^ Person-years)	*HR* (95% *CI*)^a^	*P* ^*a*^	*HR* (95% *CI*)^b^	*P* ^b^
hs-CRP		98	20,836.52	47.03	1.02 (1.00–1.05)	0.085	1.01(0.98–1.04)	0.491
	< 1.0 mg/L	37	10,951.48	33.79	2.05 (1.36–3.09)	0.001	1.54(0.99–2.36)	0.051
	≥1.0 mg/L	61	8582.37	71.08				
	< 1.08 mg/L	38	11,525.09	32.97	2.23 (1.48–3.35)	< 0.001	1.69(1.11–2.60)	0.016
	≥1.08 mg/L	60	8008.77	74.92				

*HR* hazard ratio, *CI* interval confidence

^a^Crude model

^b^Adjusted for age, gender, BMI, HDL-C, LDL-C, TC, TG, smoking, drinking, hypertension and T2DM

### Correlation of *CRP* variation with hs-CRP

*CRP* rs1205 and rs876537 C > T variations were significantly correlated with hs-CRP, after adjusted for age, gender, BMI, TC, TG, HDL-C, LDL-C, smoking, drinking, T2DM and hypertension, the standard regression coefficients were 0.064 and 0.066, respectively, *P* < 0.001. No significant correlations were observed between rs10737175, rs2808630 and hs-CRP, with *P* values of 0.713 and 0.271 (Additional file 1: Table S3). Thus, we selected rs1205 and rs876537 were selected as IVs in the MR analysis.

### MR analysis between hs-CRP and CHD

The causal effect was evaluated by using MR analysis assessing the association of genetically predicted hs-CRP with CHD risk (Table 4). The crude model showed that hs-CRP was not statistically associated with CHD, the cruder *OR* (95% *CI*) was 1.04 (0.67–1.62) with a *P* value of 0.855. The result remained negative after adjustment for age, gender, BMI, TC, TG, HDL-C, LDL-C, smoking, drinking, T2DM and hypertension, the adjust *OR* (95% *CI*) was 1.07 (0.69–2.18) with a *P* value of 0.777.

**Table 4:** The Mendelian randomization analysis of hs-CRP with CHD

Methods	*OR*	95% *CI*		*SE*	*P*
		Lower	Upper		
IVW	1.04	0.67	1.62	0.23	0.855^a^
	1.07	0.69	2.18	0.22	0.777^b^

*IVW* inverse-variance weighted, *OR* odds ratio, *CI* confidence interval, *SE* Standard error

^a^Crude model

^b^Adjusted for age, gender, BMI, HDL-C, LDL-C, TC, TG, smoking, drinking, hypertension and T2DM

## Discussion

The present study firstly applied MR design to explore the casual effect of hs-CRP and CHD risk in Chinese Han population. The main results showed that *CRP* rs1205 and rs876537 were correlated with serum hs-CRP level, but no causal effect of elevated hs-CRP on CHD risk was observed. Although the association between hs-CRP and CHD is unlikely to be causal, hs-CRP might be a predictor for incidence of CHD in general population.

Epidemiological studies have shown that plasma levels of hs-CRP are a strong independent predictor of risk of future CVDs [17, 18]. As a well-known inflammatory biomarker, hs-CRP is significantly elevated in patients dying suddenly with severe coronary artery diseases, both with and without acute coronary thrombosis [19]. There is considerable interest in establishing whether hs-CRP has a causal role in CHD. MR study, an IV-based method to infer the causality between intermediate phenotypes and disease, has been widely conducted in cardiovascular disease research [20]. Genetic variants that are associated with intermediate phenotypes are introduced as IVs in MR to estimate the effect of phenotypic exposures on disease outcome.

Previous large sample size studies showed that no association of variants in the *CRP* locus and CHD were found, arguing against a causal role for CRP in atherosclerosis [21]. Another large-scale MR research selected *CRP* rs1205 as one of IVs and indicated that CRP was unlikely to be even a modest causal factor in CHD [13]. As a non-specific inflammatory biomarker of CRP, hs-CRP takes the advantages of a higher sensitivity, accuracy and reproducibility. Our novel results firstly indicate that genetically raised hs-CRP are unrelated to the risk of CHD, which are consistent with previous studies [20, 21]. These findings imply that the cardiologists should treat CRP or hs-CRP as genuine confounders and pay more attention to other inflammatory markers in high-risk individuals.

Although American Centers for Disease Control and the American Heart Association has recommended hs-CRP 1.0 mg/L as a cut-off value to identify individuals who is under a high risk of inflammatory reaction [16], no study has proved 1.0 mg/L is applicable to Chinese population. The SROC method was used to explore the hs-CRP cut-off value when evaluating the association of CHD. Our result showed hs-CRP 1.08 mg/L is the cut-off value with a relative high sensitivity and specificity to predict CHD incidence, inferring the criterion of 1.0 mg/L is still suitable in China.

The current study investigated the cause effect of hs-CRP and CHD risk in a prospective cohort study. The strengths of this study should be noted. First, for the expensive cost of hs-CRP measurement, limited MR studies have carried out to evaluate the association of hs-CRP with CHD in a prospective cohort study. All the participants with a measurement of hs-CRP was enrolled in the follow-up study, the relative large sample size in our study substantially enhanced the statistical power, providing an objective and credible result on he-CRP and CHD. Second, it was firstly to explore the cut-off value of hs-CRP on predicting CHD incidence in Chinese Han population.

Several limitations were existed in the current study. First of all, the common SNPs that significantly correlated with hs-CRP are not derived from genome-wide association study data. In addition, the causal effect of hs-CRP on sub-types of CHD were not investigated. Besides, the pleiotropy effect of SNPs in MR analysis was not evaluated. Last but not least, during acute events, the *CRP* gene expression can be up-regulated, leading to cascades that would increase the hs-CRP levels. However, our data might not accurately represent the fundamental of gene expression related to hs-CRP.

## Conclusion

In conclusion, *CRP* rs1205 and rs876537 variation were correlated with hs-CRP level.

Although the association between hs-CRP and CHD is unlikely to be causal, hs-CRP might be a predictor for incidence of CHD in general population.

## Supplementary information

**Additional file 1.** **Table S1.** Bioinformatics Analysis of 4 selected SNPs in CRP Gene. **Table S2.** Stratification analysis of CRP gene polymorphisms and CHD. **Table S3.** Partial correlation analysis of CRP variation with hs-CRP. **Figure S1.** Survival ROC analysis for hs-CRP and CHD incidence.

## Data Availability

The datasets used and/or analyzed during the current study are available from the corresponding author on reasonable request.

## Abbreviations

CHD: Coronary heart disease
CI: Confidence interval
CVDs: Cardiovascular diseases
DL-C: High-density lipoprotein cholesterol
GLU: Glucose
HR: Hazard ratio
HRG: High risk group
Hs-CRP: High-sensitivity C-reactive protein
IV: Instrumental variable
LDL-C: Low-density lipoprotein cholesterol
MAF: Minor allele frequency
MR: Mendelian Randomization
NG: Normal group
OR: Odds Ratios
PCR: Polymerase chain reaction
SD: Standard deviation
SNP: Single nucleotide polymorphism
SROC: Survival receiver operator characteristic curve
T2DM: Type 2 diabetes mellitus
TC: Total cholesterol
TG: Triglycerides
