# Supplementary material for: Association of high sensitive C-reactive protein with coronary heart disease: a Mendelian randomization study
Source: BMC Med Genet. 2019 Nov 6;20:170. doi: 10.1186/s12881-019-0910-z (PMC6836320; doi:10.1186/s12881-019-0910-z)
Supplement: Supplementary file 1 — Additional file 1. Table S1. Bioinformatics Analysis of 4 selected SNPs in CRP Gene. Table S2. Stratification analysis of CRP gene polymorphisms and CHD. Table S3. Partial correlation analysis of CRP variation with hs-CRP. Figure S1. Survival ROC analysis for hs-CRP and CHD incidence. [file 12881_2019_910_MOESM1_ESM.docx]

**Association of High Sensitive C-reactive Protein with Coronary Heart Disease: A Mendelian Randomization Study**

Qian Zhuang^1^, Chong Shen^2^, Yanchun Chen^1^, Xianghai Zhao^1^, Pengfei Wei^1^, Junxiang Sun^1^, Yanni Ji^1^, Xiaotian Chen^3^, Song Yang^1*^

1, Department of Cardiology, Affiliated Yixing People's Hospital of Jiangsu University, Yixing, China；

2, Department of Epidemiology, School of Public Health, Nanjing Medical University, Nanjing, China.

3, Department of Clinical Epidemiology, Children's Hospital of Fudan University, Shanghai 201102, China

*Corresponding author:

Song Yang, Department of Cardiology, Affiliated Yixing People's Hospital of Jiangsu University, Yixing, TEL:0510-87330872, Email: 13921382937@163.com

TableS1. Bioinformatics Analysis of 4 selected SNPs in *CRP* Gene

| SNP | Chr | dbSNP function | Allele | TFBS | Splicing  (ESE or ESS) | Nearby Gene | MAF |
| --- | --- | --- | --- | --- | --- | --- | --- |
| rs10737175 | 1 | -- | C/T | -- | -- | CRP\|\|LOC646446 | 0.238 |
| rs1205 | 1 | 3'-UTR | C/T | -- | Y | CRP | 0.476 |
| rs2808630 | 1 | 1.2kb 3' of CRP | C/T | -- | -- | CRPP1\|\|CRP | 0.232 |
| rs876537 | 1 | -- | C/T | RP58 | -- | CRPP1 | 0.470 |

TFBS: transcription factor binding site; MAF: minor allele frequency.

Table S2: Stratification analysis of *CRP* gene polymorphisms and CHD

|  |  |  |  |  |  | HR (95% CI)^a^ | | |
| --- | --- | --- | --- | --- | --- | --- | --- | --- |
| SNP | Group | Genotype | N | Person-years | Incidence density  (/104 Person-years) | Additive model | Dominant model | Recessive model |
| rs10737175 | Male | CC | 28 | 4944.89 | 56.62 | 0.98(0.63-1.53) | 1.04(0.60-1.83) | 0.75(0.23-2.46) |
|  |  | TC | 21 | 2973.04 | 70.63 | P=0.934 | P=0.885 | P=0.640 |
|  |  | TT | 3 | 597.2 | 50.23 |  |  |  |
|  | Female | CC | 33 | 7140.24 | 46.22 | 1.13(0.71-1.79) | 1.04(0.59-1.83) | 1.75(0.63-4.89) |
|  |  | TC | 17 | 4541.89 | 37.43 | P=0.612 | P=0.890 | P=0.287 |
|  |  | TT | 4 | 649.14 | 61.62 |  |  |  |
|  | <55 years | CC | 9 | 4346.55 | 20.71 | 1.07(0.49-2.31) | 0.92(0.33-2.54) | 1.68(0.37-76) |
|  |  | TC | 5 | 2779.9 | 17.99 | P=0.869 | P=0.873 | P=0.503 |
|  |  | TT | 2 | 495.13 | 40.39 |  |  |  |
|  | ³55 years | CC | 52 | 7738.58 | 67.2 | 0.98(0.62-1.39) | 0.97(0.64-1.49) | 1.01(0.40-2.51) |
|  |  | TC | 33 | 4735.03 | 69.69 | P=0.917 | P=0.894 | P=0.990 |
|  |  | TT | 5 | 751.21 | 66.56 |  |  |  |
|  | Smoking | CC | 15 | 3012.55 | 49.79 | 1.17(0.62-2.19) | 1.11(0.52-1.38) | 1.71(0.39-7.50) |
|  |  | TC | 12 | 1831.67 | 65.51 | P=0.633 | P=0.793 | P=0.477 |
|  |  | TT | 2 | 305.1 | 65.55 |  |  |  |
|  | Non-smoking | CC | 46 | 9072.58 | 50.7 | 1.01(0.69-1.47) | 0.98(0.62-1.56) | 1.12(0.45-2.80) |
|  |  | TC | 26 | 5683.27 | 45.75 | P=0.970 | P=0.943 | P=0.806 |
|  |  | TT | 5 | 941.25 | 53.12 |  |  |  |
|  | Drinking | CC | 12 | 2587.57 | 46.38 | 0.66(0.29-1.48) | 0.74(0.29-1.88) | - |
|  |  | TC | 9 | 1675.13 | 53.73 | P=0.311 | P=0.522 | - |
|  |  | TT | 0 | 346.16 | 0 |  |  |  |
|  | Non-drinking | CC | 49 | 9497.56 | 51.59 | 1.15(0.81-1.64) | 1.09(0.71-1.69) | 1.64(0.75-3.59) |
|  |  | TC | 29 | 5839.8 | 49.66 | P=0.424 | P=0.692 | P=0.214 |
|  |  | TT | 7 | 900.18 | 77.76 |  |  |  |
| rs1205 | Male | CC | 16 | 2949.37 | 54.25 | 0.97(0.65-1.45) | 1.01(0.55-1.86) | 0.90(0.43-1.86) |
|  |  | TC | 26 | 4035.71 | 64.42 | P=0.893 | P=0.964 | P=0.770 |
|  |  | TT | 10 | 1525.05 | 65.57 |  |  |  |
|  | Female | CC | 21 | 3923.24 | 53.53 | 1.16(0.78-1.74) | 0.87(0.49-1.55) | 1.88(0.99-3.55) |
|  |  | TC | 20 | 6160.61 | 32.46 | P=0.469 | P=0.641 | P=0.053 |
|  |  | TT | 13 | 2231.34 | 58.26 |  |  |  |
|  | <55 years | CC | 7 | 2496.38 | 28.04 | 0.93(0.46-1.86) | 0.56(0.20-1.52) | 1.65(0.56-4.83) |
|  |  | TC | 4 | 3648.11 | 10.96 | P=0.829 | P=0.255 | P=0.365 |
|  |  | TT | 5 | 1471.01 | 33.99 |  |  |  |
|  | ³55 years | CC | 30 | 4376.23 | 68.55 | 1.01(0.74-1.37) | 0.94(0.60-1.48) | 1.12(0.66-1.91) |
|  |  | TC | 42 | 6548.2 | 64.14 | P=0.957 | P=0.797 | P=0.680 |
|  |  | TT | 18 | 2285.38 | 78.76 |  |  |  |
|  | Smoking | CC | 6 | 1814.89 | 33.06 | 1.23(0.70-2.17) | 1.49(0.59-3.76) | 1.14(0.43-3.05) |
|  |  | TC | 17 | 2439.81 | 69.68 | P=0.470 | P=0.397 | P=0.792 |
|  |  | TT | 6 | 894.61 | 67.07 |  |  |  |
|  | Non-smoking | CC | 31 | 5057.75 | 61.29 | 1.01(0.73-1.41) | 0.80(0.50-1.28) | 1.43(0.83-2.47) |
|  |  | TC | 29 | 7756.5 | 37.39 | P=0.947 | P=0.344 | P=0.198 |
|  |  | TT | 17 | 2861.79 | 59.4 |  |  |  |
|  | Drinking | CC | 8 | 1555.77 | 51.42 | 0.70(0.35-1.37) | 0.56(0.22-1.42) | 0.76(0.22-2.65) |
|  |  | TC | 9 | 2169.46 | 41.48 | P=0.294 | P=0.281 | P=0.671 |
|  |  | TT | 4 | 883.63 | 45.27 |  |  |  |
|  | Non-drinking | CC | 29 | 5316.84 | 54.54 | 1.17(0.85-1.60) | 1.02(0.64-1.62) | 1.53(0.91-2.57) |
|  |  | TC | 37 | 8026.86 | 46.1 | P=0.343 | P=0.926 | P=0.106 |
|  |  | TT | 19 | 2872.76 | 66.14 |  |  |  |
| rs2808630 | Male | TT | 37 | 5810.11 | 63.68 | 0.79(0.46-1.37) | 0.79(0.43-1.47) | 0.53(0.07-4.12) |
|  |  | TC | 13 | 2453.93 | 52.98 | P=0.403 | P=0.462 | P=0.539 |
|  |  | CC | 2 | 246.08 | 81.27 |  |  |  |
|  | Female | TT | 40 | 8561.8 | 46.72 | 0.87(0.49-1.52) | 0.84(0.45-1.55) | 1.03(0.14-7.53) |
|  |  | TC | 13 | 3447.43 | 37.71 | P=0.616 | P=0.576 | P=0.975 |
|  |  | CC | 1 | 317.02 | 31.54 |  |  |  |
|  | <55 years | TT | 11 | 5229.48 | 21.03 | 0.82(0.32-2.11) | 0.93(0.32-2.73) | - |
|  |  | TC | 5 | 2104.37 | 23.76 | P=0.679 | P=0.897 | - |
|  |  | CC | 0 | 287.73 | 0 |  |  |  |
|  | ³55 years | TT | 66 | 9142.43 | 72.19 | 0.78(0.51-1.20) | 0.75(0.47-1.22) | 0.73(0.16-3.23) |
|  |  | TC | 21 | 3797 | 55.31 | P=0.253 | P=0.248 | P=0.676 |
|  |  | CC | 3 | 275.36 | 108.95 |  |  |  |
|  | Smoking | TT | 17 | 3525.15 | 48.22 | 1.47(0.73-2.96) | 1.43(0.66-3.13) | 2.77(0.35-22.07) |
|  |  | TC | 10 | 1489.88 | 67.12 | P=0.281 | P=0.365 | P=0.336 |
|  |  | CC | 2 | 134.28 | 148.94 |  |  |  |
|  | Non-smoking | TT | 60 | 10846.76 | 55.32 | 0.65(0.40-1.08) | 0.63(0.37-1.08) | 0.59(0.08-4.23) |
|  |  | TC | 16 | 4411.49 | 36.27 | P=0.098 | P=0.094 | P=0.596 |
|  |  | CC | 1 | 428.81 | 23.32 |  |  |  |
|  | Drinking | TT | 13 | 3125.89 | 41.59 | 1.07(0.45-2.54) | 1.21(0.47-3.15) | - |
|  |  | TC | 7 | 1317.74 | 53.12 | P=0.876 | P=0.692 | - |
|  |  | CC | 0 | 165.23 | 0 |  |  |  |
|  | Non-drinking | TT | 64 | 11246.02 | 56.91 | 0.82(0.52-1.29) | 0.76(0.47-1.26) | 1.23(0.30-5.05) |
|  |  | TC | 19 | 4583.63 | 41.45 | P=0.384 | P=0.295 | P=0.771 |
|  |  | CC | 2 | 397.86 | 50.27 |  |  |  |
| rs876537 | Male | CC | 16 | 2916.05 | 54.87 | 0.97(0.66-1.44) | 1.04(0.57-1.90) | 0.872(0.42-1.81) |
|  |  | TC | 26 | 4043.93 | 64.29 | P=0.896 | P=0.906 | P=0.712 |
|  |  | TT | 10 | 1555.15 | 64.3 |  |  |  |
|  | Female | CC | 21 | 3933.83 | 53.38 | 1.15(0.77-1.72) | 0.87(0.43-1.53) | 1.85(0.98-3.49) |
|  |  | TC | 20 | 6145.41 | 32.54 | P=0.497 | P=0.623 | P=0.059 |
|  |  | TT | 13 | 2247.01 | 57.85 |  |  |  |
|  | <55 years | CC | 7 | 2437.26 | 28.72 | 0.91(0.45-0.1.83) | 0.54(0.20-1.49) | 1.61(0.55-4.75) |
|  |  | TC | 4 | 3697.75 | 10.82 | P=0.790 | P=0.235 | P=0.385 |
|  |  | TT | 5 | 1486.55 | 33.63 |  |  |  |
|  | ³55 years | CC | 30 | 4412.62 | 67.99 | 1.01(0.75-1.37) | 0.96(0.61-1.50) | 1.102(0.65-1.88) |
|  |  | TC | 42 | 6491.59 | 64.7 | P=0.939 | P=0.860 | P=0.723 |
|  |  | TT | 18 | 2315.61 | 77.73 |  |  |  |
|  | Smoking | CC | 6 | 1792.58 | 33.47 | 1.24(0.70-2.20) | 1.50(0.59-3.77) | 1.16(0.43-3.12) |
|  |  | TC | 17 | 2459.5 | 69.12 | P=0.456 | P=0.394 | P=0.768 |
|  |  | TT | 6 | 897.23 | - |  |  |  |
|  | Non-smoking | CC | 31 | 5057.3 | 61.3 | 1.01(0.73-1.40) | 0.80(0.50-1.29) | 1.40(0.81-2.41) |
|  |  | TC | 29 | 7729.85 | 37.52 | P=0.962 | P=0.365 | P=0.229 |
|  |  | TT | 17 | 2904.93 | 58.52 |  |  |  |
|  | Drinking | CC | 8 | 1515.04 | 52.8 | 0.68(0.35-1.35) | 0.55(0.21-1.39) | 0.74(0.21-2.57) |
|  |  | TC | 9 | 2187.27 | 41.15 | P=0.272 | P=0.204 | P=0.635 |
|  |  | TT | 4 | 906.56 | - |  |  |  |
|  | Non-drinking | CC | 29 | 5334.84 | 54.36 | 1.17(0.85-1.59) | 1.04(0.66-1.65) | 1.51(0.90-2.53) |
|  |  | TC | 37 | 8002.08 | 46.24 | P=0.336 | P=0.869 | P=0.119 |
|  |  | TT | 19 | 2895.61 | 65.62 |  |  |  |

a: Adjusted for age, gender, BMI, HDL-C, LDL-C, TC, TG, smoking, drinking, hypertension and T2DM.

Table S3. Partial correlation analysis of *CRP* variation with hs-CRP

|  |  | Hs-CRP^a^ | | |
| --- | --- | --- | --- | --- |
| SNP | Aelle | β | SD | P |
| rs10737175 | C>T | 0.006 | 0.127 | 0.713 |
| rs1205 | C>T | 0.064 | 0.110 | <0.001 |
| rs2808630 | T>C | 0.018 | 0.147 | 0.271 |
| rs876537 | C>T | 0.066 | 0.110 | <0.001 |

a: Adjusted for age, gender, BMI, HDL-C, LDL-C, TC, TG, smoking,

drinking, hypertension and T2DM.


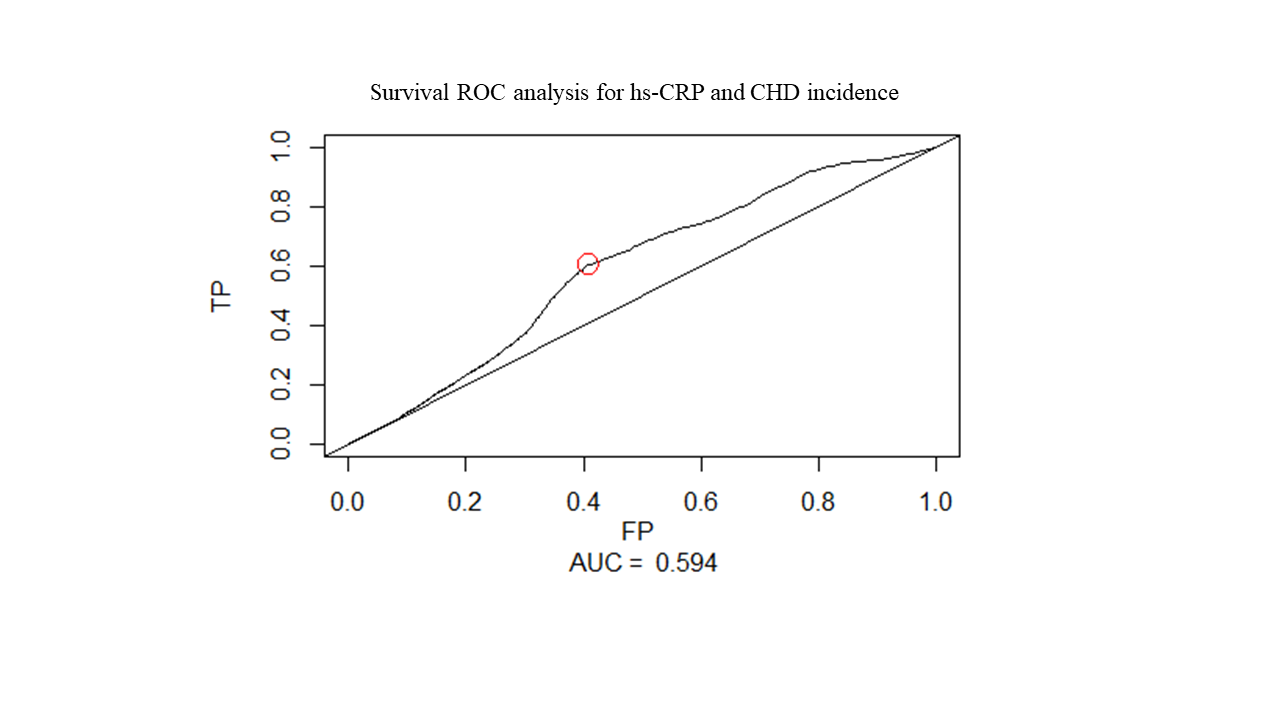


Figure S1. Survival ROC analysis for hs-CRP and CHD incidence. The survivalROC Package could estimate corresponding to the time point of interest and AUC [Area Under (ROC) Curve]) at the time point of interest. The cut-off value for hs-CRP was selected at a maximum value of TP+FP correspondingly. Red circle indicates the maximum value of TP+FP, and the corresponding cut-off value for hs-CRP is 1.08 mg/L. TP: True positive; FP: false positive.
